# Biocaiv: an integrative webserver for motif-based clustering analysis and interactive visualization of biological networks

**DOI:** 10.1186/s12859-023-05574-9

**Published:** 2023-11-29

**Authors:** Dong-Xu Li, Peng Zhou, Bo-Wei Zhao, Xiao-Rui Su, Guo-Dong Li, Jun Zhang, Peng-Wei Hu, Lun Hu

**Affiliations:** 1 The Xinjiang Technical Institute of Physics and Chemistry, Chinese Academy of Sciences, Ürümqi, China; 2 University of Chinese Academy of Sciences, Beijing, China; 3 Xinjiang Laboratory of Minority Speech and Language Information Processing, Ürümqi, China; 4 School of Computer Science and Artificial Intelligence, Wuhan University of Technology, Wuhan, China

**Keywords:** Clustering analysis, Biological networks, Bioinformatics

## Abstract

**Background:**

As an important task in bioinformatics, clustering analysis plays a critical role in understanding the functional mechanisms of many complex biological systems, which can be modeled as biological networks. The purpose of clustering analysis in biological networks is to identify functional modules of interest, but there is a lack of online clustering tools that visualize biological networks and provide in-depth biological analysis for discovered clusters.

**Results:**

Here we present BioCAIV, a novel webserver dedicated to maximize its accessibility and applicability on the clustering analysis of biological networks. This, together with its user-friendly interface, assists biological researchers to perform an accurate clustering analysis for biological networks and identify functionally significant modules for further assessment.

**Conclusions:**

BioCAIV is an efficient clustering analysis webserver designed for a variety of biological networks. BioCAIV is freely available without registration requirements at http://bioinformatics.tianshanzw.cn:8888/BioCAIV/.

## Background

In biology, many complex biological systems can be modeled as networks where individual biological entities, such as different kinds of molecules, are nodes and the connections among them are links [1, 2]. The purpose of clustering analysis in biological networks is to identify functional modules of interest, such as protein complexes and gene co-expression modules. These modules are of great significance to provide valuable insights into the understanding of corresponding biological systems [3].Therefore, many related methods and works on biological networks or clustering have been proposed [4–6].

**Fig. 1:**
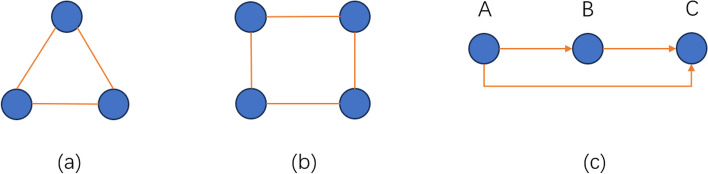
Three representative motifs in biological networks, **a** triangle motif, **b** rectangle motif and **c** the motif of feed-forward loop

Network motifs, that is, patterns of mutual connections that appear in complex networks [7]. In the context of networks, higher-order structures typically refer to the connectivity patterns involving more than just pairwise connections between nodes. Motifs are recurring and small non-random subgraphs that represent higher-order structures in a network, and their occurrences are more frequently than expected in a random network. To better illustrate the meaning of motifs, we also provide a graphical explanation in Fig. 1. We have recently presented a more general Higher-order Structural Clustering Framework (HiSCF), which aims to identify functional modules in a given biological network based on a variety of specific network motifs. HiSCF is introduced as a powerful clustering analysis tool designed for biological networks, with a focus on capturing higher-order structures, or motifs. It begins by constructing a tensor that encodes all instances of a given motif within a biological network. This step is crucial for capturing the structural characteristics of the motif. To address the clustering problem, HiSCF formulates it as a higher-order Markov chain model, which is computationally intractable. Hence, it leverages the concept of spacey random walk theory to approximate the higher-order Markov chain by a first-order Markov chain. The Markov Clustering Algorithm is then employed by using the transition matrix of the first-order Markov chain, allowing for the effective identification of clusters specific to the network motif under consideration [8]. However, the implementation of HiSCF integrates several complex programming environments python, Julia, and java, thus limiting its wide applicability. The compatibility issue is also difficult to address for non-programmer biological researchers who are not familiar with Python, Julia or Java. It is for this reason that we would like to develop an online framework that facilitate the implementation and usage of HiSCF in a concise and reproducible manner.

Currently, only few web tools have been made available to the scientific community on the task of clustering analysis for biological networks. They are either in the form of plugins or web-based. For example, GraphWeb [9] is a public web server developed for discovering functional modules through biological network analysis. To improve the clustering efficiency, SPICi [10] is dedicated as a web tool to provide fast clustering analysis for large biological networks. ClusterViz [11] is designed as a plugin package of Cytoscape to conduct the clustering analysis of biological networks. To our knowledge, no new online clustering tools have emerged recently for biological networks. In particular, none exists for visualizing biological networks and providing an in-depth analysis for discovered clusters.

In this context, we present a more comprehensive and user-friendly web server, namely BioCAIV, which integrates HiSCF to offer motif-based clustering analysis for biological networks. BioCAIV is composed of three modules: (1) a backend service to reimplement HiSCF, (2) a web interface to upload network files and present clustering results, (3) a visualization tool able to visualize the input network, and (4) a functional enrichment analysis service to investigate the functional correlation of molecules in the clusters.

Currently, BioCAIV offers two distinct operational modes: the Normal Mode and the Custom Mode, each catering to specific network characteristics and user preferences. In the Normal Mode of BioCAIV, the platform is designed to support undirected networks. This limitation arises from the input format of network data, which does not accommodate directional information. For users who require the flexibility to work with both directed and undirected networks, BioCAIV offers the Custom Mode. In this mode, the platform provides comprehensive support for both network types. Users can process directed and undirected networks within BioCAIV, provided that all motif instances extracted from the biological network are correctly formatted in the input file.

BioCAIV is a promising tool capable of performing clustering analysis on a wide range of biological networks. However, there are specific considerations when it comes to functional enrichment analysis, depending on the nature of the biological network. First of all, BioCAIV is capable of conducting clustering tasks on a variety of biological networks, regardless of their types. For biological networks such as protein–protein interaction networks, gene regulatory networks, and gene co-expression networks, users can leverage BioCAIV’s clustering capabilities for these network types without restrictions. Secondly, functional enrichment analysis within BioCAIV is specifically tailored for biological networks composed of genes, or gene-coded proteins. Hence, biological networks containing gene-related nodes, such as protein–protein interaction networks, gene regulatory networks, and gene co-expression networks, are eligible for functional enrichment analysis. Residue interaction networks, characterized by nodes representing individual protein residues, are not suitable for functional enrichment analysis provided by g:Profiler, as their nodes do not involve gene-level entities.

This new web server addresses the drawbacks of HiSCF as it is easy to use with a web interface. Specifically, when compared with HiSCF, BioCAIV is preferred from several aspects. First, it can be easily accessed through internet without compiling sources codes. Second, unlike HiSCF that only supports triangle and rectangle motifs, it allows users to perform clustering analysis for any higher-order structures they are interested in. Last, we also provide the results of functional enrichment analysis, thus enabling users to refine identified modules that are functionally significant and should be experimentally verified for potential applications. In particular, there are several distinct advantages to using BioCAIV in comparison to other programs. These advantages make BioCAIV a highly attractive tool for users in the field of bioinformatics, and they are listed as below.

1. *User-Friendly Accessibility*: BioCAIV eliminates the need for users to possess specialized knowledge on compiling source code written in various programming languages. Unlike standalone programs like HiSCF, BioCAIV offers an accessible and hassle-free experience for researchers, regardless of their programming expertise.
2. *Seamless Computational Resources*: Traditional standalone clustering software, like Cytoscape, often rely on the computational power of users’ local computers. In contrast, BioCAIV centralizes all necessary computational resources on its dedicated server. This approach ensures consistent and efficient performance, irrespective of the user’s hardware capabilities.
3. *Advanced Clustering Technique and User-Centric Features*: BioCAIV distinguishes itself from existing online clustering tools in two significant ways. Firstly, it employs a state-of-the-art clustering algorithm, resulting in more accurate and insightful analyses. Secondly, it provides a range of user-friendly functions, including email notifications, functional enrichment analysis, and network visualization. These features enhance the overall usability and productivity of the platform.

In light of these compelling advantages, we firmly believe that BioCAIV stands as an valuable tool for the clustering analysis of biological networks.

## Implementation

**Fig. 2:**
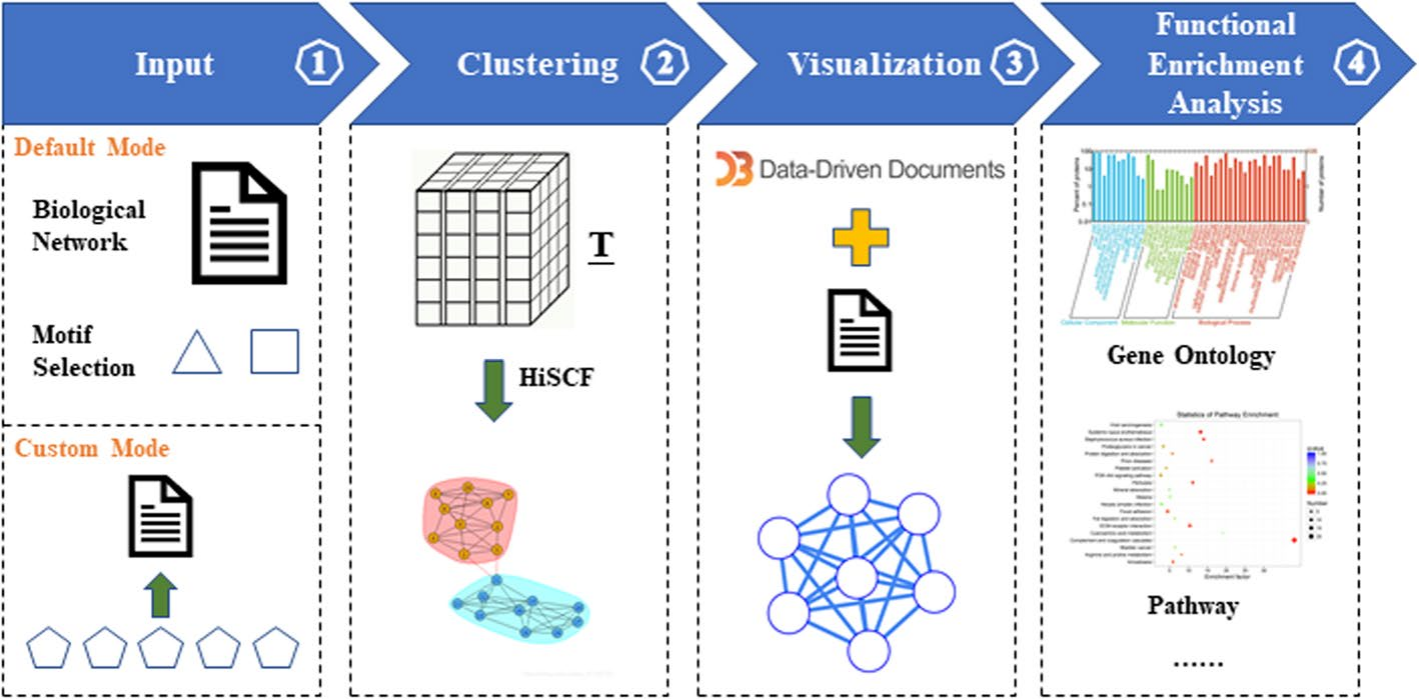
Overview of the BioCAIV pipeline. Step 1. Input is provided in the formatted file that specifies details on the biological network to be analyzed. Step 2. A tensor-based variable is constructed to apply HiSCF for discovering clusters. Step 3. BioCAIV makes use of D3.js to fastly visualize the input network with interactive functions. Step 4. Clustering results are further analyzed to search functionally significant clusters

As a modular web server, BioCAIV is more advanced than existing clustering tools by providing three features enabling it to visualize and analyze biological networks, and search functionally significant clusters. The overall framework of BioCAIV is presented in Fig. 2.

### Input module

**Fig. 3:**
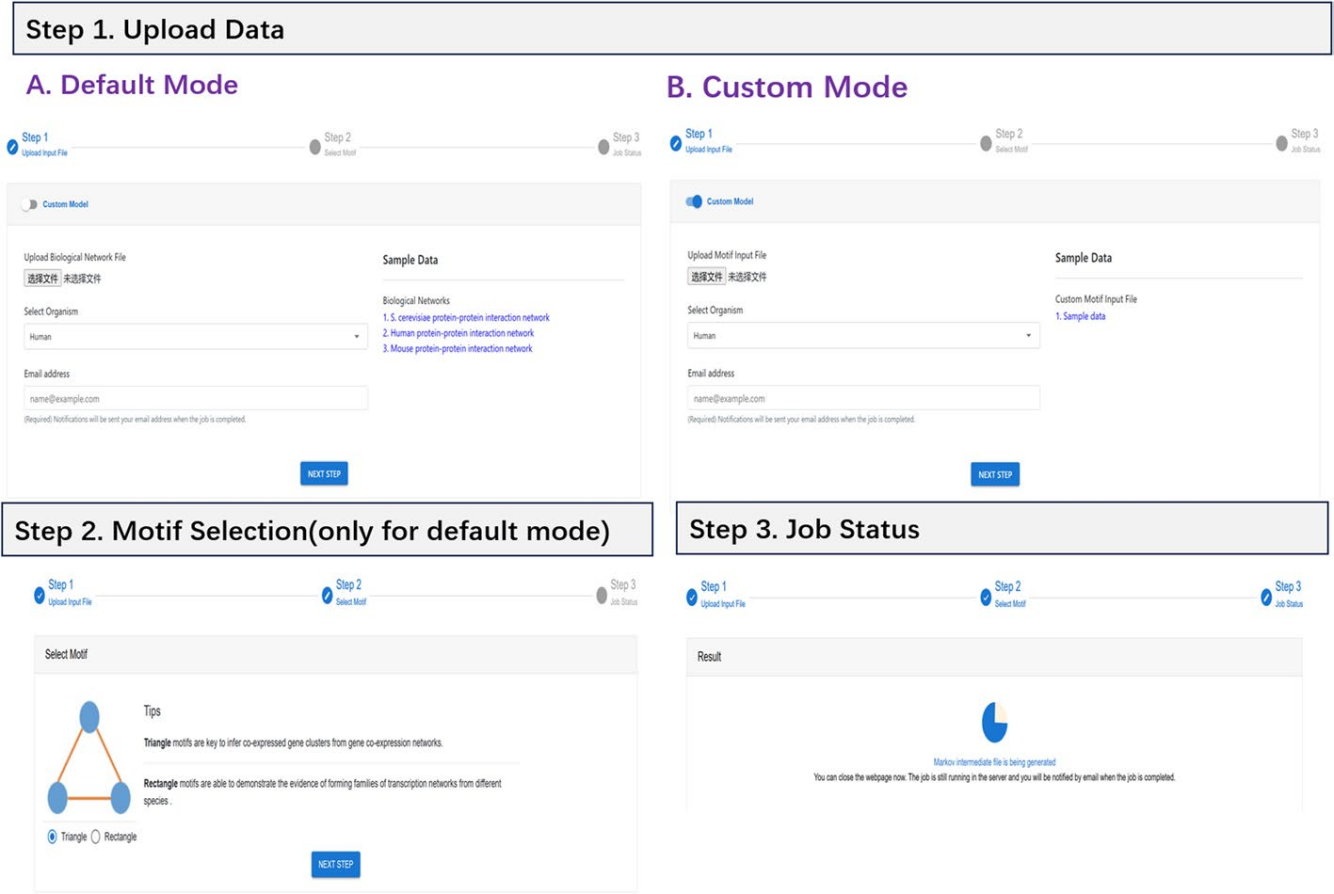
The workflow of input module

BioCAIV currently operates in two modes. One is *Default* mode where two kinds of motifs, i.e., Triangle and Rectangle, are supported by default. The other is *Custom* mode where clustering analysis can be performed on any particular motif specified by users. To use BioCAIV, users are required to input biological networks or motif instances corresponding to different modes by specifying the following information. The workflow of input module is presented in Fig. 3.

1. Default Mode
  Biological Network File: the file can be uploaded via BioCAIV. Three sample network files are provided as an illustration to the data format. They are, S. cerevisiae protein–protein interaction network, Human protein–protein interaction network and Mouse protein-protein interaction network. Incorrect biological network files will lead to the failure of clustering analysis. With respect to the input format, BioCAIV utilizes a straightforward and distinctive representation. Two specific letters, ’v’ and ’e’, are employed to indicate nodes and edges, respectively, within the input file. In particular, rows beginning with ’v’ are used to denote node identifiers. These ’v’-prefaced rows contain information about individual nodes within the network. Rows that start with ’e’ are employed to describe the connections between pairwise nodes. These ’e’-prefaced rows specify the relationships or edges between nodes in the network.
  Motif Selection: users need to select a motif, either Triangle or Rectangle, used for clustering analysis.
2. Custom Mode
  Motif Input File: the file is uploaded via BioCAIV. Users have to create this file based on the motif they are interested in. A sample motif file is provided to describe the data format. In particular, each line is composed of nodes separated by tab and it is an instance of that particular motif in the biological network.

One should note that in the either biological network or motif input files, molecules have to be represented by their corresponding Entrez Gene IDs if functional enrichment analysis is applied. In addition, the following information should be provided by users for both modes.

- Organism Selection: currently BioCAIV supports Three organisms, i.e., Human, Saccharomyces Cerevisiae and Mouse for functional enrichment analysis. More organisms will be included upon the requests of users.
- Email: once the job is submitted, a link to the job status page will be sent to the user. Moreover, a notification will also be sent to the user when the job is completed.The algorithm takes a certain amount of time to run. During the running time, there is no need to wait on the page all the time. You can close the page to check the prompt email in the email and click the “View Results” button to jump to the result page.

Upon successful submission of the input data, users are redirected to the job page, where the current status is presented and the results will be displayed upon completion.

### Visualization module

In the post-genomics era, biological networks have been becoming increasingly larger. Their visualization can be troublesome and time-consuming, thus requiring efficient and easy-to-use interfaces to present their topological structures in an autonomous manner. In BioCAIV, we visualize the input biological network by using D3.js [12], which is a JavaScript library having full capabilities of modern browsers without installing any plugins. Due to the minimal overhead taken by D3.js, BioCAIV is able to support the visualization of large biological networks with fast interactive behaviors.

### Clustering analysis module

Before performing the clustering analysis, BioCAIV first defines a tensor variable to indicate all instances of the selected motif. Taking the triangle motif as an example, a three-mode tensor is defined as ${{{\underline{\varvec{T}}}}}=\big (\underline{t}(i,j,k)\big )$. Regarding the value of $\underline{t}(i,j,k)$, we have $\underline{t}(i,j,k)=1$ if nodes $v_i$, $v_j$ and $v_k$ construct a triangle, and $\underline{t}(i,j,k)=0$ otherwise. BioCAIV then follows the procedure of HiSCF to identify clusters upon ${{{\underline{\varvec{T}}}}}$.

**Fig. 4:**
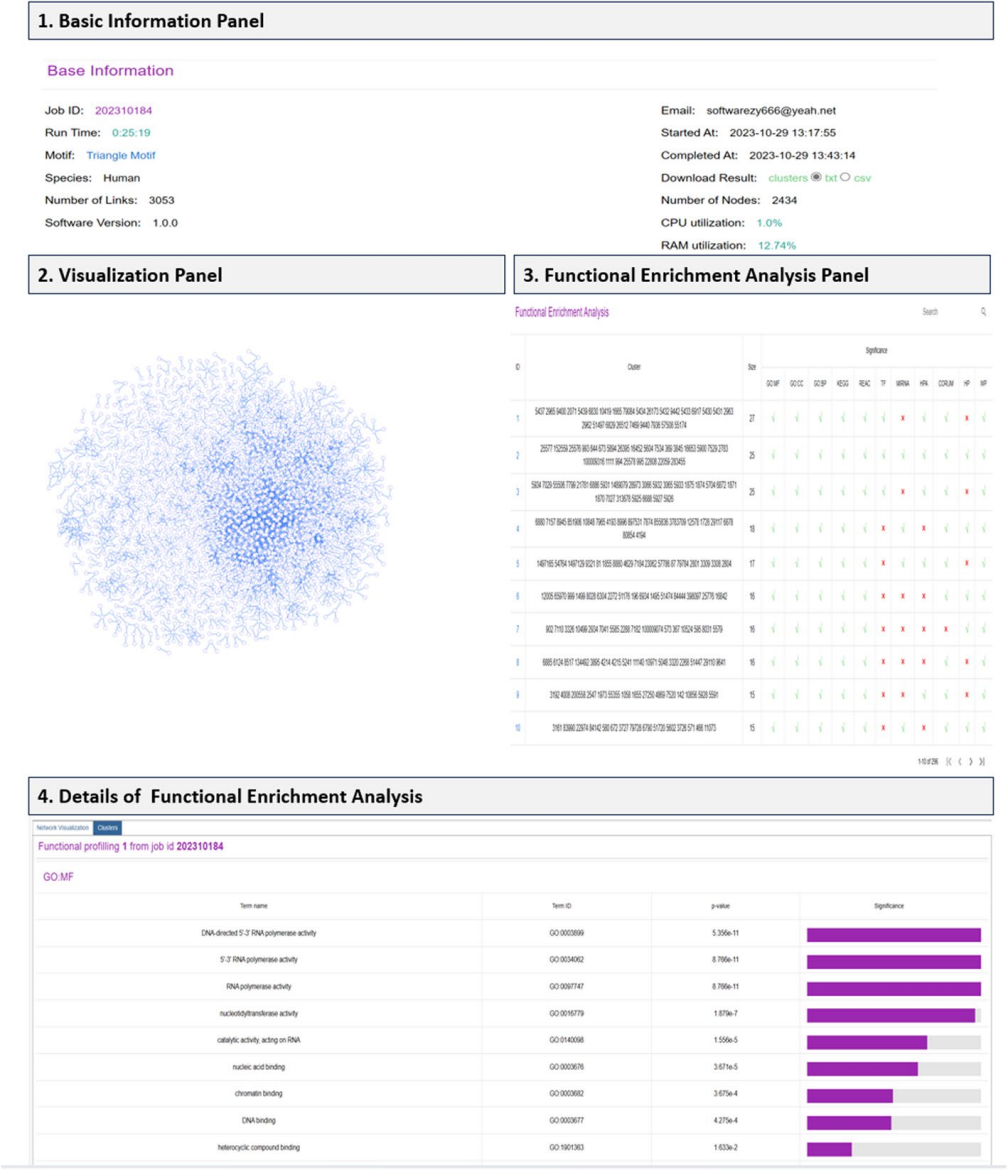
BioCAIV provides (1) basic information panel of the task, (2) the visualization of input biological network, (3) the overall picture of functional enrichment analysis of all clusters, and (4) the details of functional enrichment analysis for each cluster

The clustering analysis job is usually completed less than half an hour for a biological network composed of thousands of nodes. Even users close the webpage, the job is still running in the server side and users will be notified by email when the job is completed. The clustering results are composed of three panels displaying the following information. An overview of the output page is reported in Fig. 4.

- Basic Information. In addition to Job ID and Email address, we also provide the time information for the job. The clustering result is available to download by clicking the “clusters” link. we have included additional metrics in the result page. Alongside the running time, the average usage of CPU and RAM resources is now available. This information offers a more detailed perspective on the computational demands of the analysis.
- Network Visualization. The input biological network is visualized in this panel, which also allows users to interact with the network.
- Clusters. In this panel, we list the discovered clusters as well as their component nodes. Besides, we also present the overall assessment regarding the functional significance of clusters.

### Output file

The output file generated by BioCAIV adopts a simplified structure. Each row in the output file comprises node identifiers separated by spaces. These rows effectively represent functional modules or clusters identified by BioCAIV during the clustering analysis. In addition to the plain text output format, we have introduced an enhanced output format: CSV (Comma-Separated Values). This CSV format is designed for compatibility with various data analysis applications, including popular tools like Cytoscape, ensuring seamless integration for downstream analysis.

### Functional enrichment analysis module

In this module, users can retrieve the result of functional enrichment analysis, which is driven by Python script of g:Profiler [13], for each cluster. In particular, BioCAIV additionally provides functional enrichment analysis for identified modules from different resources of biological information, including functional categories from Gene Ontology [14], pathways from WikiPathways [15], regulatory motif matches from TRANSFAC [16] and human disease phenotypes from Human Phenotype Ontology [17]. In the panel of functional enrichment analysis, an overall picture is presented to indicate the significance of all clusters under different biological information. By clicking the cluster ID, significant terms identified by g:profiler in each resource are displayed for the corresponding cluster. When BioCAIV retreives the functional enrichment analysis from g:profiler, the network interruption may occur. To overcome this issue, BioCAIV first popups a message box showing the text “Failed to retrieve the results of functional enrichment analysis from g:profiler. Reload or not?” If users face the data loading problem. Once the OK button is clicked, BioCAIV will reload the current page to retrieve the results from g:profiler again.

### Use of case

In particular, we take the sample data of Human protein–protein interaction network as the input biological network. Since feed-forward loops are neither triangle nor rectangle motifs, we opt to select the Custom Model. Then a customized input file should be prepared for providing the feed-forward loops in the network. In this file, each row represents a unique feed-forward loop extracted from the network, and it should consist of node identifiers that constitute that loop and are separated by tabs. Upon uploading the customized input file, users will be prompted to input their email addresses. This is essential to ensure that users receive timely notifications regarding the processing status of their job. Once users click “Next Step,” they will be redirected to the result page. This page provides real-time information about the status of the current job, allowing users to monitor the progress of their analysis. In addition to that, we set up the “Functional enrichment analysis on disease analysis” example on the Use of Case page. In this case study, we take the clustering results, obtained from Human protein–protein interaction network by using the Rectangle Motif, as an example to provide disease analysis through functional enrichment analysis.

### The organization of BioCAIV

As for BioCAIV, the *Home* page is organized with a top banner displaying several available sections. Dedicated tabs help users easily navigate to browse the content of each section. Moreover, it also describes the general information of BioCAIV and presents the input form to initialize a new clustering analysis task. It is also possible to run a test job by using the sample data provided in *Home*.

Besides *Home*, the *Contact*, *References* and *Help* pages are also provided. Explanations regarding the usage of BioCAIV and the required input formats are reported in *Manual*. The implemented clustering algorithm, i.e., HiSCF, is introduced in *Method*. The *References* page provides the relevant literature related to BioCAIV and the *Help* page provides links to feedback the problems encountered by users as well as their suggestions on this web tool.

The BioCAIV web server is written in Python 3.7 based on the Tornado framework. The server is containerized with Virtualvenv and deployed on a dedicated GNU/Linux server.

## Results and discussion

In BioCAIV, the integration of tensor-based data structures and efficient clustering algorithm allows users to study the higher-order organization patterns in biological networks. On the one hand, BioCAIV simplifies the clustering analysis by offering easy-to-use functionalities, and also creates an intuitive interface to visualize the biological networks. BioCAIV renders a variety of motifs into a uniform analysis pipeline, with which clusters can thus be identified upon a specific motif. On the other hand, looking at the topological structures of clusters may admittedly fail to provide valuable insights into biological systems. To overcome this problem, BioCAIV allows researchers to approach a number of functional enrichment analysis tasks from different biological information perspectives. Functionally significant clusters are worth further investigation. The main advantage of BioCAIV is its simple interface and fast speed in visualizing and handling large biological networks for clustering analysis. Hence, we have reason to believe that BioCAIV is a useful web server in discovering and analyzing clusters from biological networks, since it allows users to reduce the analysis complexity. Such a reduction may broaden the feasibility of BioCAIV to more potential applications in the field of bioinformatics.

Regarding the future work, we would like to unfold it from four aspects. First, BioCAIV currently provides two motifs, i.e., triangle and rectangle, for clustering analysis, and more motifs will be implemented in *Default* mode to facilitate the usage of BioCAIV. Second, two organisms are supported by BioCAIV to perform functional enrichment analysis on discovered clusters, and we are interested in converting more organisms in future. Third, more interactive operations will be incorporated into BioCAIV when we visualize the biological networks as well as discovered clusters. Such an improvement may provide access to more topological analysis methods. Last, we also would like to explore more potential applications by using BioCAIV, such as drug repositioning [18] and miRNA-disease association prediction [19].

## Conclusions

We have presented here a novel web server, namely BioCAIV, that allows users to perform an accurate clustering analysis for biological networks by using the higher-order structural information provided by users. As an extension of HiSCF, this web server needs only biological network file or custom motif file, not requiring any additional data and it is preferred for clustering analysis over HiSCF due to its user-friendly interface and free availability. Thus, its applicability will be maximized by targeting to a large audience, especially for biological researchers with limited computational resources. Moreover, with the creation of BioCAIV, we wish to be dedicated to addressing the clustering tasks for understanding the functional mechanisms of different biological networks, and providing a tool by emphasizing its ease of use.

## Data Availability

Project name: BioCAIV. Project home page: http://bioinformatics.tianshanzw.cn:8888/BioCAIV/. Operating system(s): Platform independent. Programming language: Python. Other requirements: Python 3.7 or higher. License: GNU GPL. Any restrictions to use by non-academics: No restrictions.

## Abbreviation

HiSCF: Higher-order structural clustering framework
